# Factors associated with fear avoidance beliefs among University students with Low Back Pain in the United Kingdom: A cross-sectional survey study protocol

**DOI:** 10.1371/journal.pone.0323955

**Published:** 2025-05-20

**Authors:** Tsit Yu Serena Chow, Chinonso Nwamaka Igwesi-Chidobe

**Affiliations:** 1 School of Allied Health Professions and Midwifery, Faculty of Health Studies, University of Bradford,; 2 Global Population Health (GPH) Research Group, University of Nigeria, Nsukka, Nigeria; Iran University of Medical Sciences, IRAN, ISLAMIC REPUBLIC OF

## Abstract

Low back pain (LBP) is the leading cause of chronic disability and is predicted to escalate in the next 20 years globally. Fear Avoidance Beliefs (FAB) are one of the most important factors associated with low back pain outcomes, including the first onset of back pain, pain intensity, pain episodes, disability and quality of life. With the prevalence rate of low back pain in young adults increasing exponentially, the factors associated with fear avoidance beliefs among university students in the United Kingdom (UK) are currently unknown. This study, therefore, aims to investigate the factors associated with fear avoidance beliefs among UK university students with low back pain. An online cross-sectional study will be conducted. University students with low back pain in the UK will be recruited through university contacts and social media of the 131 universities in the UK. The online survey will collect demographic information (sex, age, ethnicity, religion, the regional location of the university, the faculty the participant is currently studying in, working status and hours of work), perceived cause of LBP, severity of pain, pain site and symptoms, pain duration, previous surgeries in the lower back area, treatment received for low back pain, the experience of seeing family members/ significant others with disabling low back pain, advice received from others, physical activity levels, mental health status and disability levels, and fear avoidance beliefs will be collected. Descriptive analysis (frequencies and percentages, means and standard deviations, median and interquartile range) will summarise data. Correlation analysis will be used to assess bivariate associations between variables. Multiple linear regression analysis will determine the factors associated with fear avoidance beliefs.

## Introduction

### Background

Low Back Pain (LBP) occurs in the lumbosacral area, between the 12th rib and the iliac crest, and sometimes to the buttock and gluteal folds. It can be a dull ache or sharp pain and may radiate into other areas of the body, especially the legs [1–3]. LBP is divided into acute (<4 weeks), subacute (4 weeks to 3 months) and chronic (>3 months) [4]. LBP is the leading cause of chronic disability, affecting 619 million people globally and explicitly affecting more females than males [5]. Prediction reports suggest that LBP cases will continue to increase by at least 25% between 2020 and 2050 due to population growth and ageing [5].

The prevalence of LBP in young adults has been increasing, which is the typical age of university students [6]. The prevalence rate of LBP among young adults has increased exponentially over the decades [5]. It is suggested that by age 20, more than 50% of males and females had experienced at least one episode of LBP [7]. Studies worldwide have shown that university students have a high prevalence rate. However, the prevalence rate of the types of LBP and sexes varies between studies, possibly due to geographical differences [8–12]. For example, research in Saudi Arabia [8] showed that lifetime prevalence was more common and that males had a higher prevalence rate among health science students. On the other hand, a study in Austria [10] showed that chronic LBP has the lowest prevalence rate and a similar rate between the two sexes. However, other studies in Australia, China, Ethiopia, France, Hong Kong, Hungary, India, Serbia, Singapore, Sweden, the United States, and Saudi Arabia [9,12] showed that females had a higher prevalence rate.

Fear Avoidance Beliefs (FAB) are the beliefs that performing movements and doing work is harmful and should be avoided and include pain perception & emotional reaction [13,14]. It suggested that people would fall into the spectrum of confrontation or avoidance. People who fall towards confrontation will likely have adaptive responses like viewing pain as a temporary issue, are strongly motivated to return to everyday lifestyles, and are ready to confront pain until it is resolved or undergo conservative management or surgery accordingly [13–15]. On the other hand, people who avoid pain experiences and expect painful movements will fall towards being less adaptive in response to pain caused by fear [13,15]. High fear-avoidance beliefs may cause activity levels to decrease due to pain, which influences recovery potentials and outcomes [14,16,17].

FAB have been found to be one of the most important factors associated with LBP outcomes, including the first onset of back pain, pain intensity, pain episodes, disability, and quality of life globally in high, middle, and low-income countries [18,25,27–30]. Results from studies in Nigeria revealed that fear avoidance beliefs are associated with many LBP outcomes including pain episodes, self-reported and performance-based disability [28,29]. Notably, in this population, FAB were associated with most neck pain outcomes, including pain intensity, neck disability and sick leave [30]. These results mirror the results from high-income countries like the United States [31].

Current research suggests that factors like sex, levels of disability, occupation, physical activity levels, mental health, advice received, experiences of seeing others with disabling LBP, and previous surgeries are associated with the FAB of patients with chronic LBP [18–23]. A study [24] also suggested that Physiotherapy students tend to have more positive beliefs than Medical and Nursing students when it comes to chronic LBP, and females are more likely to be negative. A study in France [25] suggested that high physical-demand jobs, disability perception, low education levels, and negative physician beliefs increase subacute LBP patients’ FAB. The factors associated with acute LBP are similar to chronic LBP, with the addition of working status. Although patients’ FAB tend to decrease after four weeks as many LBP are self-limiting, knowing the level of FAB and the factors with this would help identify patients with a higher risk of prolonged disability and how to mitigate these [19,20,26]. It is suggested that university students with LBP have prolonged sitting issues due to studying, with the prevalence rate of LBP increasing along with their year of study [32].

A study in Japan [18] investigated the factors associated with FAB among people with LBP. 52,650 participants aged 20–79 with LBP completed the questionnaire they created. Information like demographic data, weight, height, and experience of LBP were collected, along with other factors like regular exercise, smoking habits, working status, marital status, history of lower back surgery, advice, etc. Disability levels were graded from 0 to 4, ranging from no pain to having more than four consecutive days of absences from social activities. Results showed that all variables were relevant to FAB except for smoking habits and marital status, and the experience of LBP was the most relevant to the FABQ-J physical activity score. However, this research included an extensive age range. It did not further explore the influence of educational factors on LBP, where education was simply separated into completed or not completed college or others.

There is limited literature on FAB among general university students. Most research only focused on university students studying healthcare courses like Nursing, Medicine, Dentistry, and Physiotherapy or did not mention the courses the students undertook [8–12,33]. Moreover, most studies explored the factors associated with FAB in chronic LBP, with limited research also including those with acute and subacute LBP. Furthermore, limited studies have been conducted in the United Kingdom (UK). Therefore, the factors associated with FAB among general university students with acute, subacute and chronic LBP in the UK require further investigation. This study (Master of Physiotherapy research project) aims to investigate the factors associated with FAB amongst general university students in the UK with any type of LBP (acute, subacute or chronic LBP). The Strengthening the Reporting of Observational Studies in Epidemiology (STROBE) statement was used to report this study [34].

### Objectives

1. Investigate the factors (sociodemographic variables, site and symptoms, pain frequency and duration, severity of pain, perceived cause of LBP, previous surgeries in the lower back area, the experience of seeing family members/ significant others with disabling LBP, advice received from others, working status and hours, physical activity levels and mental health status) associated with Fear Avoidance Beliefs among general University students in the UK with acute, subacute or chronic non-specific LBP using correlation analyses.
2. Identify which of these factors with significant correlations in correlation analyses would predict fear avoidance beliefs in the multiple linear regression model.

## Materials and methods

### Study design and setting

An online cross-sectional survey will be conducted with students in UK universities. There are over 130 universities across the UK [35], and all universities will be included in this study to achieve the full coverage of UK university students. Data collection through the online survey began on 16th October 2024 and will be closed on 1st April 2025. Results will be expected by 28th April 2025. Data collection is currently 87% completed.

### Ethical considerations

Ethical approval was obtained from the University of Bradford’s Humanities, Social, and Health Sciences Research Ethics Panel (E1249).

Additional information regarding the ethical, cultural, and scientific considerations specific to inclusivity in global research is included in the Supporting Information (S1 Checklist)

### Participants

Convenience sampling and volunteer opt-in sampling methods will be used in this study [36,37], in which data collection is currently in progress and will continue until March 2025. An online cross-sectional survey will be made available to the public via the distribution of website links. Participants will be recruited from both universities’ emails and through social media. The communications departments, press departments and student unions of the 131 universities will be contacted via email and direct messages through their social media handles (Instagram, Facebook, LinkedIn, and X) to seek involvement and distribute the questionnaire to the targeted population. The researcher and supervisor will also distribute the survey link via their social media accounts. By clicking on the survey link and submitting the survey, students will be recruited and seen as participants in this study. Due to the use of the convenience sampling method, the sample’s representativeness of the population will be less optimal than a probability sampling method. To ameliorate this, a longer time for data collection will assist in recruiting a larger sample size from all UK universities following a clear and strict inclusion and exclusion criteria to ensure representativeness of the population [38].

The Jisc Online Survey website was used to create the questionnaire and collect participant data. Before filling in the questionnaire, participants must read the information sheet explaining the questionnaire process. The researcher’s and supervisor’s contact information are also available on the sheet for any queries. Then, participants will need to confirm that they understood the purpose of the study and are willing to participate in this study voluntarily by filling in the informed consent form. The information sheet and consent form clearly state that the questionnaire is voluntary to complete, participants may contact the mental health services (Samaritans and Shout) given on the information sheet if they feel uncomfortable during or after the completion of survey, all data will be destroyed after the study has been completed and analysed, the results of the survey will be presented in data form, participants can choose not to complete or submit the questionnaire, and all incomplete and unsubmitted data will not be saved, participants may request to withdraw their survey anytime permanently after completion without any given reason, and that the survey is anonymous and fully confidential. Consent forms are shown after the information sheet, by clicking ‘yes’ on the consent form, the survey will automatically document the consent given.

Three screening questions must be filled in to confirm that participants are eligible for the study. The questionnaire automatically excludes participants if they are not university students (undergraduate/ postgraduate/ pre-registration course/ post-registration course), if they have not experienced LBP in the past four weeks, or if LBP is related to menstruation, feverish illness, any congenital or acquired musculoskeletal deformity, underlying serious pathology, pregnancy-related or pelvic inflammatory diseases [39], and they have participated in similar studies previously. Participant’s responses will not be recorded if they were excluded due to not meeting eligibility criteria.

### Variables and measurement

The questionnaire (S1 Appendix) consists of four sections (excluding consent and three screening questions): demographic information, information on LBP and possible factors associated with FAB, the Fear Avoidance Beliefs Questionnaire (FABQ), and the Oswestry Disability Index (ODI). All questions will be answered by multiple-choice, rating, or short answer. The questions are based on suggested factors associated with FAB from previous literature.

In section one, there are six questions to collect the general demographic characteristics of the participants, including sex, age, ethnicity, religion, the regional location of the university, and the faculty the participant is currently studying.

The second section consists of 12 questions that need to be filled in for the information and possible factors related to FAB, including site and symptoms, pain frequency and duration in multiple-choice questions, and severity of pain on a Likert scale of 1–10 [39]. The perceived cause of LBP, previous surgeries in the lower back area, treatment received for LBP, the experience of seeing family members/ significant others with disabling LBP, advice received from others, working status and hours, physical activity levels and mental health status will also be asked in the format of multiple choice. Sub-questions may need to be filled in depending on the participant’s answer to collect further details within specific factors.

This survey uses the Beck Depression Inventory (BDI) and Beck Anxiety Inventory (BAI) to further understand participants’ mental health status. Both self-assessment tools are commonly used for screening and measuring depression and anxiety levels in psychiatry with high sensitivity and specificity [40–44]. The BDI consists of 21 categories of symptoms and attitudes in depression. Each question will have four answers that will score from 0–4, and the participants will choose the answer most applicable to them [40,41]. Total score of 0–9 as minimal depression, 10–18 as mild depression, 19–29 as moderate depression and 30–63 as severe depression. Similarly, the BAI also consist of 21 items with the same scoring system. With the total score 0–7 as minimal anxiety, 8–15 as mild anxiety, 16–25 moderate anxiety and 26–63 as severe anxiety [42–44].

The 16-item FABQ in the third section, consists of 5 questions about physical activity (FABQ-PA) and 11 questions about work-related activity (FABQ-W) that would be answered on a scale of 0–6 [45]. Scores from each question will be added together, with a minimum score of 0 and a maximum score of 96. The higher the total score, the higher the level of FAB. It is a tool used to identify and assess participants’ beliefs about the impact of physical activity and occupation on their LBP. Studies showed that the FABQ is both valid and reliable; it is also an excellent prognostic tool for LBP, which aids in increasing the attention to address the issue with FAB earlier on [45–47].

The ODI is in the fourth section of the questionnaire. It is a tool to help indicate the level of function in daily activities for those with LBP. It consists of 10 questions relating to everyday activities: pain intensity, personal care, lifting, walking, sitting, standing, sleeping, social, travel, and employment and homemaking. Each question has six statements to choose from and is scored from 0 to 5 [48]. The lower total score would indicate a lower level of disability in LBP and vice versa, with the possible lowest score of 0 and the highest score of 50. The use of ODI is because it has a high reliability and validity for non-specific LBP with questions that will be able to match participants’ daily activities [17,49]. Although it will not be included in the regression model, it will used (as a continuous variable) to further understand the characteristics of participants by understanding their disability levels.

### Bias control

Exposure bias would be minimised by screening out participants who had been involved in similar research previously [50]. Recall bias would be minimised by restricting the previous experience of LBP to four weeks [39]. Sampling bias will be reduced by trying to distribute questionnaire links to all UK universities through a range of social media sources and emails [51].

### Study size

A priori sample size calculation was performed with G Power version 3.1.9.6 software [52]. The sample size of 222 would give 95% power to detect a medium regression effect size (f2) of 0.15 at alpha (α) of 0.05 with tested predictors of 20. This is the maximum number of variables that we assume will have significant correlations with fear avoidance beliefs.

## Statistical methods

Data will be analysed using the Statistical Package for Social Sciences (IBM SPSS), version 28.0. Descriptive analysis (frequencies and percentages; means and standard deviations; median and interquartile range) will be used to summarise data. Bivariate analysis including correlation tests will be used to assess associations between each variable and fear avoidance beliefs [53]. When any of these variables (sociodemographic variables, site and symptoms, pain frequency and duration, severity of pain, perceived cause of LBP, previous surgeries in the lower back area, the experience of seeing family members/ significant others with disabling LBP, advice received from others, working status and hours, physical activity levels and mental health status) demonstrate a statistically significant bivariate correlation with fear avoidance beliefs, they will be included in the multiple linear regression model. Multiple linear regression analysis will then determine the factors associated with fear avoidance beliefs by determining the unique variance contributed by each significant predictor [54].

## Limitations

As the survey was updated when data collection had started, data on treatment received for LBP and mental health status will be affected. Therefore, these two variables may not have the same number of participants as other variables. However, data collection will continue until these variables are adequately powered.

## Supporting information

S1 Appendix Study questionnaire. (DOCX)
