# Supplementary material for: Factors associated with fear avoidance beliefs among University students with Low Back Pain in the United Kingdom: A cross-sectional survey study protocol
Source: PLoS One. 2025 May 20;20(5):e0323955. doi: 10.1371/journal.pone.0323955 (PMC12091721; doi:10.1371/journal.pone.0323955)
Supplement: S1 Appendix — (DOCX) [file pone.0323955.s001.docx]

# S1 Appendix: Study Questionnaire


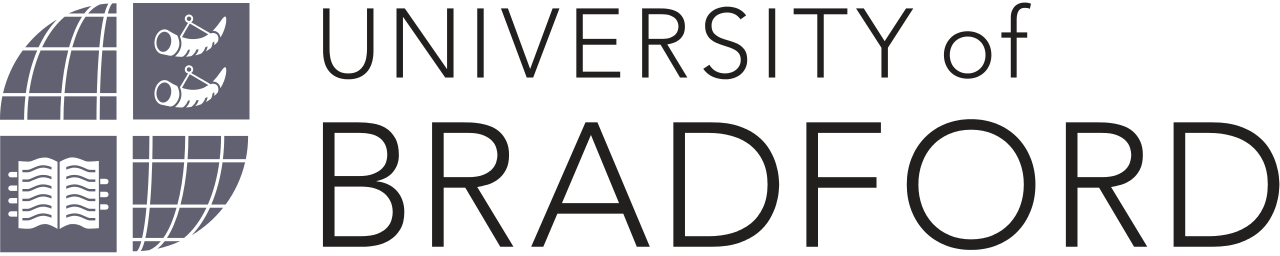


**Survey on Factors Associated with Fear Avoidance Beliefs among University Students in the UK with Low Back Pain**

# Introduction

Hi,

I am Serena Chow, a final year student studying MPhysiotherapy Sport and Exercise Medicine) in the

University of Bradford. I am currently performing a research to investigate the factors associated with Fear Avoidance Beliefs among University students in the UK with Low Back Pain for my Dissertation, and would like to invite you to participate in this research project.

This questionnaire consist of multiple choice, rating and short answer questions, which will take approximately 15 minutes to complete. This questionnaire is voluntary to complete, and you may decide not to complete the survey or not submit the survey after starting it without any explanation, all uncomplete survey will not be saved.

Submitted data will all be stored in the Jisc Survey Website and will be destroyed once all data had been analysed. Data can be deleted sooner and permanently if you request to.

Please consider participating in this study if you are a University student (undergraduate/ postgraduate/ pre-registration course/post-registration course) studying in the UK and have experienced non-specific low back pain in the last four weeks.

You will not be eligible for this research if you are not a university student, not studying in the UK and have not experienced non-specific low back pain in the past four weeks, or if your low back pain is related to menstruation or feverish illness.

This study had obtained ethical approval from University of Bradford E1249

If you have any queries regarding to this research, please contact the researcher below for more information before completing the survey. Contact information:

Researcher: Miss Tsit Yu Serena Chow

Email: t.y.s.chow@bradford.ac.uk

Supervisor: Dr. Chinonso Igwesi-Chidobe

Email: c.igwesi-chidobe@bradford.ac.uk

If you feel distressed after completing this survey, you may contact the mental health service suggested below.

1. Samaritans Phone Number (free from any phone, available 24/7 116 123 Email: jo@samaritans.org
2. Shout 85258 Text SHOUT to 85258 (free and available 24/7

By clicking Next at the bottom of the screen, you will be directed to the consent form, after the completion of the consent form will you then be lead to the screening questions and the questionnaire.

Thank you very much for your participation!

# Informed Consent

 I confirm that I understood the purpose of the study and consider to participate in this study voluntarily with sufficient time to consider and ask questions about the research.

 I understand that the questionnaire may involve sensitive/personal questions that may cause some psychological discomfort; if I feel distressed after completing it, I may contact the mental health service provided on the information sheet.

 I understand that all submitted data will be stored in the Jisc Survey Website and will be destroyed once all data had been analysed.

 I understand that I can request to withdraw my survey anytime after completion without any given reason.

 I understand that the survey is anonymous and fully confidential. Also understand that I can contact the researcher/ researcher's supervisor for further details regarding to this study.

 I understand that the result of this survey would be used for the study and presented in data form.

## I have read the statement above and provide consent to participate in this research. *

Yes

No

## Are you a University Student Undergraduate/ Post-graduate/ pre-registration course/post-registration course) studying in the United Kingdom? *

Yes

No

**Have you experienced Non Specific Low Back Pain in the past 4 weeks? Excluding Pain from menstruation, feverish Illness, any congenital or acquired musculoskeletal deformity such as kyphosis, lordosis, scoliosis, and kyphoscoliosis or any underlying serious pathology including malignancy, infection, fracture, spinal stenosis, metabolic disorders, or pregnancy-related or due to pelvic inflammatory diseases) ***

Yes

No

## Have you been involved in similar studies previously? *

Yes

No

# Demographics

## Sex *

Male

Female

## Age *

## Ethinicity *

Asian or Asian British  Inidian

Asian or Asian British  Pakistani

Asian or Asian British  Bangladeshi

Asian or Asian British  Chinese

Black, Black British, Caribbean or African  Caribbean

Black, Black British, Caribbean or African  African

Mixed or multiple ethnic groups  White and Black Caribbean

Mixed or multiple ethnic groups  White and Black African

Mixed or multiple ethnic groups  White and Asian

White  English, Welsh, Scottish, Northern Irish or British

White  Irish

White  Gypsy or Irish Traveller

White  Roma

Other  Arab

Other

**If you selected Other, please specify: ***

## Religion *

Christian

Buddhism

Muslim

Islam

Hinduism

No religious beliefs

Other

## If you selected Other, please specify: *

## Which region is your University located? *

North East

North West

Yorkshire and the Humber

East Midlands

East of England

West Midlands

London

South East

South West

Scottland

Wales

Northern Ireland

## Faculty *

Medicine, Health Studies & Healthcare Business, Management, Finance & Economics

Life Science & Natural Science

Humanities & Social Science

Law

Engineering

Art & Design

Other

## If you selected Other, please specify: *

# Low Back Pain

## Does your Low Back Pain travel down the leg? *

Yes

No

## If yes, does the pain spread below the knee? *

Yes

No

## Frequency of Low Back Pain *

3 days per week

3 days per week

Everyday

## How long have you had the issue of Low Back Pain for? *

6 weeks

6 weeks but 12 weeks

12 weeks

## Rate your severity of Low Back Pain *

### Very Low

1

2 3 4

5 6

7

8 9

10

## Cause(s) of Low Back Pain *

Non-Specific

Occupational

Traffic Accident

Sports Injury

Specific Disease

## Name of Disease *

## Have you had any previous surgery for your Low Back Pain? *

Yes

No

## Have you had any experience of seeing family member(s) and/or significant others with disabling Low Back Pain? *

Yes

No

## Have you received any advice from others about your Low Back Pain? *

Yes

No

## What were the type of advice(s) given? *

Resting

Posture

Lifting

Other

## If you selected Other, please specify: *

### Have you received any specific medical intervention for your low back pain? *

Yes No

**If chose yes, please specify the medical intervention.**

## Have you been exercising regularly? At least 150300 minutes of moderate-intensity aerobic exercise or 75150 minutes of vigorous-intensity aerobic exercise or equivalent combination of moderate and vigorous-intensity activity) *

Yes

No

### What is your current working status? (including both paid and unpaid internship) *

Working

Not working

## Type of work *

Heavy Lifting Labour

Light Lifting Labour

Mostly Sitting

Mostly Standing

Mostly Walking

Other

## If you selected Other, please specify: *

## Working Hours *

25 hours per week

25 hour per week but 40 hours per week

40 hours per week

## Do you have any Mental Health Disorder(s)? *

Yes

No

## What is your Mental Health Disorder(s)? *

Depression

Anxiety

Other

## If you selected Other, please specify: *

# Beck's Depression Inventory Questionnaire

Choose the most applicable answer.

*****

1. I do not feel sad.
2. I feel sad.
3. I am sad all the time and I can't snap out of it.
4. I am so sad and unhappy that I can't stand it.

*****

1. I am not particularly discouraged about the future.
2. I feel discouraged about the future.
3. I feel I have nothing to look forward to.
4. I feel the future is hopeless and that things cannot be done.

*****

1. I do not feel like a failure.
2. I feel I have failed more than the average person.
3. As I look back on my life, all I can see is a lot of failures.
4. I feel I am a complete failure as a person.

*****

1. I get as much satisfaction out of things as I used to.
2. I donʼt enjoy things the way I used to.
3. I don't get real satisfaction out of anything anymore.
4. I am dissatisfied or bored with everything.

*****

1. I donʼt feel particularly guilty.
2. feel guilty a good part of the time.
3. I feel quite guilty most of the time.
4. I feel guilty all of the time.

*****

1. I donʼt feel I am being punished.
2. I feel I may be punished
3. I expect to be punished.
4. I feel I am being punished.

*****

0 I donʼt feel disappointed in myself 1 I am disappointed in myself.

1. I am disgusted with myself.
2. I hate myself.

*****

1. I donʼt feel I am any worse than anybody else.
2. I am critical of myself for my weakness or mistakes.
3. I blame myself all the time for my faults.
4. I blame myself for everything bad that happens.

*****

1. I donʼt have any thoughts of killing myself.
2. I have thoughts of killing myself, but I would not carry them out.
3. I would like to kill myself.
4. I would kill myself if I had the chance.

*****

donʼt cry any more than usual.

I cry more now than I used to.

2 I cry all the time now.

3 I used to be able to cry , but now I canʼt cry even though I want to.

*****

1. I am no more irritated by things that I ever was.
2. I am slightly more irritated now than usual.
3. I am quite annoyed or irritated a good deal of the time.
4. I feel irritated all the time.

*****

1. I have not lost interest in other people.
2. I am less interested in other people than I used to be.
3. I have lost most of my interest in other people.
4. I have lost all of my interest in other people.

*****

1. I make decisions about as well as I ever could.
2. I put off making decisions more than I used to.
3. I have greater difficulty in making decisions more than I used to.
4. I can't make decisions at all anymore.

*****

1. I donʼt feel that I look any worse than I used to.
2. I am worried that I am looking old or unattractive.
3. I feel there are permanent changes in my appearance that make me look unattractive.
4. I believe that I look ugly.

*****

can work about as well as before.

It takes an extra effort to get started at doing something.

2 I have to push myself very hard to do anything 3 I can't do any work at all.

*****

1. I can sleep as well as usual.
2. I donʼt sleep as well as I used to.
3. I wake up 12 hours earlier than usual and find it hard to get back to sleep.
4. I wake up several hours earlier than I used to and cannot get back to sleep.

*****

1. I don't get more tired than usual.
2. I get tired more easily than I used to.
3. I get tired from doing almost anything.
4. I am too tired to do anything.

*****

0 My appetite is no worse than usual 1 My appetite is not as good as It used to be 2 My appetite is much worse now.

3 I have no appetite at all anymore.

*****

1. I haven't lost much weight, if any, lately.
2. I have lost more than five pounds.
3. I have lost more than ten pounds.
4. I have lost more than fifteen pounds.

*****

am no more worried about my health than usual

I am worried about physical problems like aches, pain, upset stomach or constipation.

1. I am very worried about physical problems and it's hard to think of much else.
2. I am so worried about my physical problems that I cannot think of anything else.

*****

1. I have not noticed any recent changes in my interest in sex.
2. I am less interested in sex than I used to be.
3. I have almost no interest in sex.
4. I have lost interest in sex completely.

# Beck Anxiety Inventory Questionnaire

Choose the most applicable answer.

## Choose the most applicable answer according to the past month. *

### Numbness or tingling

1. Not at all
2. Mildly, but itdidnʼt botherme much
3. Moderately – itwasnʼt pleasantat times
4. Severely – itbothered me a lot

### Feeling hot

1. Not at all
2. Mildly, but itdidnʼt botherme much
3. Moderately – itwasnʼt pleasantat times
4. Severely – itbothered me a lot

### Wobbliness in legs

1. Not at all
2. Mildly, but itdidnʼt botherme much
3. Moderately – itwasnʼt pleasantat times
4. Severely – itbothered me a lot

### Unable to relax

1. Not at all
2. Mildly, but itdidnʼt botherme much
3. Moderately – itwasnʼt pleasantat times
4. Severely – itbothered me a lot

### Fear of worst happening

1. Not at all
2. Mildly, but itdidnʼt botherme much
3. Moderately – itwasnʼt pleasantat times
4. Severely – itbothered me a lot

### Dizzy or lightheaded

1. Not at all
2. Mildly, but itdidnʼt botherme much
3. Moderately – itwasnʼt pleasantat times
4. Severely – itbothered me a lot

### Heart pounding / racing

1. Not at all
2. Mildly, but itdidnʼt botherme much
3. Moderately – itwasnʼt pleasantat times
4. Severely – itbothered me a lot

### Unsteady

1. Not at all
2. Mildly, but itdidnʼt botherme much
3. Moderately – itwasnʼt pleasantat times
4. Severely – itbothered me a lot

### Terrified or afraid

1. Not at all
2. Mildly, but itdidnʼt botherme much
3. Moderately – itwasnʼt pleasantat times
4. Severely – itbothered me a lot

### Nervous

1. Not at all
2. Mildly, but itdidnʼt botherme much
3. Moderately – itwasnʼt pleasantat times
4. Severely – itbothered me a lot

### Feeling of choking

1. Not at all
2. Mildly, but itdidnʼt botherme much
3. Moderately – itwasnʼt pleasantat times
4. Severely – itbothered me a lot

### Hands trembling

1. Not at all
2. Mildly, but itdidnʼt botherme much
3. Moderately – itwasnʼt pleasantat times
4. Severely – itbothered me a lot

### Shaky / unsteady

1. Not at all
2. Mildly, but itdidnʼt botherme much
3. Moderately – itwasnʼt pleasantat times
4. Severely – itbothered me a lot

### Fear of losing control

1. Not at all
2. Mildly, but itdidnʼt botherme much
3. Moderately – itwasnʼt pleasantat times
4. Severely – itbothered me a lot

### Difficulty in breathing

1. Not at all
2. Mildly, but itdidnʼt botherme much
3. Moderately – itwasnʼt pleasantat times
4. Severely – itbothered me a lot

### Fear of dying

1. Not at all
2. Mildly, but itdidnʼt botherme much
3. Moderately – itwasnʼt pleasantat times
4. Severely – itbothered me a lot

### Scared

1. Not at all
2. Mildly, but itdidnʼt botherme much
3. Moderately – itwasnʼt pleasantat times
4. Severely – itbothered me a lot

### Indigestion

1. Not at all
2. Mildly, but itdidnʼt botherme much
3. Moderately – itwasnʼt pleasantat times
4. Severely – itbothered me a lot

### Faint / lightheaded

1. Not at all
2. Mildly, but itdidnʼt botherme much
3. Moderately – itwasnʼt pleasantat times
4. Severely – itbothered me a lot

### Face flushed

1. Not at all
2. Mildly, but itdidnʼt botherme much
3. Moderately – itwasnʼt pleasantat times
4. Severely – itbothered me a lot

### Hot / cold sweats

1. Not at all
2. Mildly, but itdidnʼt botherme much
3. Moderately – itwasnʼt pleasantat times
4. Severely – itbothered me a lot

# Fear Avoidance Belief Questionnaire

0 Completely Disagree

6 Completely Agree

## My pain is caused by physical activity

### 0 Completely Disagree 6 Completely Agree *

#### Completely Disagree

0

1

2 3 4 5

6

## Physical activity makes my pain worse

### 0 Completely Disagree 6 Completely Agree *

#### Completely Disagree

0

1

2 3 4 5

6

## Physical activity might harm my back

### 0 Completely Disagree 6 Completely Agree *

#### Completely Disagree

0

1

2 3

4

5

6

**I should not do physical activities which (might) make my pain worse**

### 0 Completely Disagree 6 Completely Agree *

#### Completely Disagree

0

1

2 3 4 5

6

**I cannot do physical activities which (might) make my pain worse**

### 0 Completely Disagree 6 Completely Agree *

#### Completely Disagree

0

1

2 3 4 5

6

## My pain was caused by my work or by an accident at work

### 0 Completely Disagree 6 Completely Agree *

#### Completely Disagree

0

1

2 3 4 5

6

## My work aggravated my pain

### 0 Completely Disagree 6 Completely Agree *

#### Completely Disagree

0

1

2 3 4 5

6

**I have a claim for compensation for my pain**

### 0 Completely Disagree 6 Completely Agree *

#### Completely Disagree

0

1

2 3 4 5

6

## My work is too heavy for me

### 0 Completely Disagree 6 Completely Agree *

#### Completely Disagree

0

1

2

3 4 5

6

## My work makes or would make my pain worse

### 0 Completely Disagree 6 Completely Agree *

#### Completely Disagree

0

1

2 3 4 5

6

## My work might harm my back

### 0 Completely Disagree 6 Completely Agree *

#### Completely Disagree

0

1

2 3 4 5

6

**I should not do my normal work with my present pain**

### 0 Completely Disagree 6 Completely Agree *

#### Completely Disagree

0

1

2 3 4 5

6

**I cannot do my normal work with my present pain**

### 0 Completely Disagree 6 Completely Agree *

#### Completely Disagree

0

1

2 3 4 5

6

**I cannot do my normal work till my pain is treated**

### 0 Completely Disagree 6 Completely Agree *

#### Completely Disagree

0

1

2 3 4 5

6

**I do not think that I will be back to my normal work within 3 months**

### 0 Completely Disagree 6 Completely Agree *

#### Completely Disagree

0

1

2 3 4 5

6

**I do not think that I will ever be able to go back to that work**

### 0 Completely Disagree 6 Completely Agree *

#### Completely Disagree

0

1

2 3 4 5 6

# Oswestry Low Back Disability Questionnaire

Please choose the most suitable description.

## Pain Intensity *

### Choose the most suitable description

I can tolerate the pain I have without having to use painkillers

The pain is bad but I manage without taking painkillers

Painkillers give complete relief from pain

Painkillers give moderate relief from pain

Painkillers give very little relief from pain

Painkillers have no effect on the pain and I do not use them

## Personal Care (e.g. Washing, Dressing) *

### Choose the most suitable description

I can look after myself normally without causing extra pain

I can look after myself normally but it causes extra pain

It is painful to look after myself and I am slow and careful

I need some help but manage most of my personal care

I need help every day in most aspects of self care

I donʼt get dressed, I was with difficulty and stay in bed

## Lifting *

### Choose the most suitable description

I can lift heavy weights without extra pain

I can lift heavy weights but it gives extra pain

Pain prevents me from lifting heavy weights off the floor, but I can manage if they are conveniently positioned, i.e. on a table

Pain prevents me from lifting heavy weights, but I can manage light to medium weights if they are conveniently positioned

I can lift very light weights

I cannot lift or carry anything at all

### Walking *

#### Choose the most suitable description

Pain does not prevent me walking any distance

Pain prevents me walking more than one mile

Pain prevents me walking more than ½ mile

Pain prevents me walking more than ¼ mile

I can only walk using a stick or crutches

I am in bed most of the time and have to crawl to the toilet

### Sitting *

#### Choose the most suitable description

I can sit in any chair as long as I like

I can only sit in my favorite chair as long as I like

Pain prevents me from sitting more than one hour

Pain prevents me from sitting more than ½ hour

Pain prevents me from sitting more than 10 minutes

Pain prevents me from sitting at all

### Standing *

#### Choose the most suitable description

I can stand as long as I want without extra pain

I can stand as long as I want but it gives me extra pain

Pain prevents me from standing for more than one hour

Pain prevents me from standing for more than 30 minutes

Pain prevents me from standing for more than 10 minutes

Pain prevents me from standing at all

### Sleeping *

#### Choose the most suitable description

Pain does not prevent me from sleeping well

I can sleep well only by using medication

Even when I take medication, I have less than 6 hrs sleep Even when I take medication, I have less than 4 hrs sleep

Even when I take medication, I have less than 2 hrs sleep

Pain prevents me from sleeping at all

### Social Life *

#### Choose the most suitable description

My social life is normal and gives me no extra pain

My social life is normal but increases the degree of pain

Pain has no significant effect on my social life apart from limiting my more energetic interests, i.e. dancing, etc.

Pain has restricted my social life and I do not go out as often

Pain has restricted my social life to my home

I have no social life because of pain

### Travelling *

#### Choose the most suitable description

I can travel anywhere without extra pain

I can travel anywhere but it gives me extra pain

Pain is bad, but I manage journeys over 2 hours

Pain restricts me to journeys of less than 1 hour

Pain restricts me to short necessary journeys under 30 minutes

Pain prevents me from traveling except to the doctor or hospital

### Employment/Homemaking *

#### Choose the most suitable description

My normal homemaking/ job activities do not cause pain.

My normal homemaking/ job activities increase my pain, but I can still perform all that is required of me.

I can perform most of my homemaking/ job duties, but pain prevents me from performing more physically stressful activities (e.g. lifting, vacuuming)

Pain prevents me from doing anything but light duties.

Pain prevents me from doing even light duties.

Pain prevents me from performing any job or homemaking chores.
